# Post-mortem detection of six human herpesviruses (HSV-1, HSV-2, VZV, EBV, CMV, HHV-6) in trigeminal and facial nerve ganglia by PCR

**DOI:** 10.7717/peerj.6095

**Published:** 2019-01-09

**Authors:** Iwona Ptaszyńska-Sarosiek, Justyna Dunaj, Agata Zajkowska, Anna Niemcunowicz-Janica, Monika Król, Sławomir Pancewicz, Joanna Zajkowska

**Affiliations:** 1 Department of Forensic Medicine, Medical University of Bialystok, Białystok, Poland; 2 Department of Infectious Diseases and Neuroinfections, Medical University of Bialystok, Białystok, Poland; 3 Department of Neurology, Medical University of Bialystok, Bialystok, Poland

**Keywords:** Herpesvirus, Latency, Trigeminal, Facial, Ganglia, HHV

## Abstract

**Background:**

Among over 100 types of *Herpesviridae* viruses, eight can infect humans: herpes simplex viruses (HSV-1, HSV-2), varicella zoster virus (VZV), cytomegalovirus (CMV), Epstein-Barr virus (EBV), and human herpesviruses 6, 7, and 8 (HHV-6, HHV-7, HHV-8). After initial infection, the viruses remain latent for the lifetime of the host. The aim of this study was to determine the distribution of six different herpesviruses: HSV-1, HSV-2, VZV, EBV, CMV, and HHV-6 in trigeminal and facial nerve ganglia among a random group of Polish population.

**Methods:**

The studied group consisted of 47 individuals (40 male, seven female); mean age of 47.4 ± 16.5 years) who died of independent causes (suicide, traffic accident, and poisoning, among others). Bilateral trigeminal and facial nerve ganglia of each cadaver were collected during the autopsy. Herpesviruses were detected using multiplex polymerase chain reaction technique.

**Results:**

Herpesviruses were found in trigeminal and/or facial ganglia in 30/47 (63.8%) of cadavers. HHV-6 was the most prevalent of the herpesviruses and was found in nearly half of cadavers (*n* = 22; 46.8%), followed by HSV-1 (*n* = 7; 14.9%), VZV (*n* = 4; 8.5%), EBV (*n* = 4; 8.5%), HSV-2 (*n* = 2; 4.3%), and CMV (*n* = 1; 2.1%). Facial nerve ganglia (*n* = 23; 48.9%) were more often infected than trigeminal ganglia (*n* = 13; 27.7%).

**Discussion:**

The results of this study have revealed a common presence of the herpesviruses in trigeminal and facial nerve ganglia among a random group of Polish population. Furthermore, the data also demonstrate simultaneous infection of the ganglia with different herpesviruses. This study has contributed to the knowledge of prevalence and localization of herpesviruses in different structures of the nervous system.

## Introduction

There are over 100 known herpesviruses, among which eight infect humans: herpes simplex viruses type 1 and 2 (HSV-1 and HSV-2), varicella zoster virus (VZV), cytomegalovirus (CMV), Epstein-Barr virus (EBV), and human herpesviruses 6, 7, and 8 (HHV-6, HHV-7, HHV-8). Following primary infection, herpesviruses enter a dormant state and remain in the host for a lifetime (Kinchington et al., 2012). It has been reported that most of the human herpesviruses establish latency by forming an extrachromosomal episome, repressing viral replication and limiting gene expression. However, the exact mechanism varies depending on a virus, for example, HHV-6A integrates into the telomeric region of host chromosomes rather than forming episomes (Pantry & Medveczky, 2017). Herpesviruses are divided into three subfamilies (α, β, γ) based on their biological properties and tropism for different types of host cells. The alphaherpesviruses (HSV-1, HSV-2, VZV) stay dormant in neurons, gammaherpesviruses (EBV, HHV-8) establish latency within immune system cells (mainly B-cells) and betaherpesviruses (CMV, HHV-6) remain latent in hematopoietic cells (White, Beard & Barton, 2012; Bloom, Giordani & Kwiatkowski, 2010). Most of the initial infections are asymptomatic and occur in childhood (White, Beard & Barton, 2012; Bloom, Giordani & Kwiatkowski, 2010). Persistence and latency of alphaherpesviruses have been well demonstrated in human trigeminal, facial, and vestibular ganglia. Reactivation of the HSV in these ganglia has been associated with various disorders such as herpes labialis, Bell’s palsy and vestibular neuritis (Theil et al., 2002). Furthermore, it has also been postulated that the virus may be spread from trigeminal ganglia to brain parenchyma and produce encephalitis (Bradshaw & Venkatesan, 2016). VZV reactivation may have various manifestations including shingles (herpes zoster) with classic vesicular rash, dermatomal pain syndromes without rash (zoster sine herpete), neuropathy or even encephalitis (Gilden et al., 2010). EBV, CMV, and HHV-6 may also have neurotropic potential and have been implicated in numerous neurological conditions, such as meningitis, encephalitis, cranial, or peripheral neuritis. The possible association between primary malignancies of central nervous system and herpesviruses has also been described (Strojnik, Duh & Lah, 2017; Meyding-Lamadé & Strank, 2012). Herpesviruses can reactivate in both immunocompetent and immunocompromised hosts. However, the risk of some life-threatening complications, such as disseminated zoster due to VZV reactivation or lymphoproliferative disease caused by EBV is greater among immunocompromised individuals (Malaiya et al., 2015). The most important immune defense against viral infections is the cell-mediated response with a crucial role of T-cells. Alterations in T-cell function and level are of particular relevance for latent viruses reactivation. The cellular immunity, in contrast to the humoral one, decreases with age, giving the opportunity for virus reactivation in elderly people (White, Beard & Barton, 2012). As most humans are supposedly infected with multiple herpesviruses, the risk of virus reactivation and possible complications, especially if immunosuppression occurs, is significant. Multiple studies have concentrated on the presence of HSV-1, HSV-2, and VZV in cranial nerve ganglia, but there is limited data about the occurrence of EBV, CMV, and HHV-6 in these ganglia (Saitoh et al., 2013; Motani et al., 2006; Richter et al., 2009; Hill et al., 2008; Vrabec & Payne, 2001; Inoue et al., 2010; Cohrs, Barbour & Gilden, 1996; Furuta et al., 1992, 1997; Hüfner et al., 2007). The aim of this study was to determine the distribution of six different herpesviruses: HSV-1, HSV-2, VZV, EBV, CMV, and HHV-6 in trigeminal and facial nerve ganglia of the individuals who died of independent causes.

## Material and methods

The studied group consisted of 47 individuals (40 male, seven female; mean age of 47.4 ± 16.5 years) who died due to independent reasons (suicide, traffic accident, and poisoning, among others). The study was approved by the Bioethical Committee of the Medical University of Bialystok (R-I-002/360/2015). The material used in the study was collected as a part of a mandatory panel of samples secured during forensic autopsy in the Forensic Medicine Department at the Medical University of Bialystok. Informed consent from the next of kin for autopsy and collection of the samples was not required due to obligatory nature of the forensic autopsy. Bilateral trigeminal and facial nerve ganglia were collected during the autopsy and divided into small fragments. Samples were stored in sterile eppendorfs and frozen at −20 °C.

### Methods—DNA extraction

DNA was extracted by using the Qiagen DNAeasy Blood and Tissue Mini kit with spin columns according to manufacturer’s instructions. Purified DNA isolates (200 µl) of left and right particular cranial nerve were combined in one probe and were frozen at −20 °C before the time of amplification.

### Methods—Herpesviruses (HSV-1, HSV-2, VZV, EBV, CMV, HHV-6) PCR detection

For simultaneous HSV-1, HSV-2, VZV, EBV, CMV, and HHV-6 molecular detection *The Seeplex Meningitis-V1 ACE Detection [V2.0] PCR kit* (Seegene, Seoul, Korea) for in vitro diagnostic was used (IVD CE mark). Highly conservative genes of HSV-1, HSV-2, VZV, EBV, CMV, and HHV-6 were simultaneously detected using one-tube multiplex PCR method. The kit used in the study is based on a concept of oligo technology—dual priming oligonucleotide (DPO^™^, Seegene, Seoul, South Korea) technology. DPO technology maximizes PCR specificity and sensitivity and optimizes amplification by fundamentally blocking non-specific priming. For control of nucleic acid extraction procedure and to check eventual inhibition of PCR amplification in clinical samples collected from patients, Internal Control (IC) was introduced into each amplification reaction and was co-amplified with target nucleic acid from the specimen. Addition of the 8-methoxypsoralen (MOP-8) was used to extinguish the template activity of contaminated DNAs and eliminate carry-over contamination.

Amplification was performed on the SensoQuest LabCycler (SensoQuest, Göttingen, Germany) in compliance with *Seegene* instructions. PCR Mastermix contained 10 μl of Multiplex Master Mix (DNA polymerase and buffer with dNTPs), two μl of MOP-8, two μl of 10× MB ACE PM (Primer Mixture—primer pairs for six viral pathogens and for IC), and one μl of IC. A five μl of the template DNA extract from nerve ganglia was added to 15 μl of the Mastermix for final reaction mix volume of 20 μl. The course of the amplification was prepared according to the following reaction program: initial denaturation at 94 °C for 15 min, amplification for 40 cycles: denaturation at 94 °C for 30 s, annealing at 63 °C for 90 s, extension at 72 °C for 90 s, and final extension at 72 °C for 10 min.

During 120 min of electrophoresis at 90 V, amplification products were separated on 2% agarose gel (Sigma-Aldrich, Darmstadt, Germany) containing Midori Green DNA Stain (Nippon Genetics, Tokyo, Japan). Amplicons were visualized by means of UV illumination in Gel Logic 100 Imaging System (Kodak, Rochester, NY, USA). Positive samples were those with amplification products with the length of 189 bp for HSV-1, 308 bp for HSV-2, 251 bp for VZV, 400 bp for EBV, 590 bp for CMV, 482 bp for HHV-6. Additionally, 1,000 bp long fragments of internal standard were detected in all samples. Results were compared with positive control (mixture of six viruses clones) provided in the detection kit. Negative control (sterilized water) which replaced template DNA was also provided by the manufacturer. Molecular weight markers (M100-M500, M100-1000-Blirt S.A. Poland), as well as MV2ACE Marker (provided), were used for each electrophoresis phase on an agarose gel to identify the approximate size of the target product.

## Results

Herpesviruses were detected in trigeminal and/or facial nerve ganglia in 30/47 (63.8%) of cadavers. HHV-6 was the most prevalent of herpesviruses and was found in nearly half of cadavers (*n* = 22; 46.8%) in analyzed samples, followed by HSV-1 (*n* = 7; 14.9%), VZV (*n* = 4; 8.5%), EBV (*n* = 4; 8.5%), HSV-2 (*n* = 2; 4.3%), and CMV (*n* = 1; 2.1%). Herpesviruses were detected in 23/47 (48.9%) samples of facial nerve ganglia and 13/47 (27.7%) of trigeminal ganglia. In the trigeminal ganglia of three cadavers, simultaneous presence of multiple herpesviruses was found: EBV/HHV-6 in one case and VZV/HHV-6 in two cases. Moreover, dual infection was also found in five samples of facial nerve ganglia: HSV-1/HHV-6 in two cases and isolated cases of HSV-2/EBV, VZV/CMV, and EBV/HHV-6 co-presence. Simultaneous occurrence of different herpesviruses was found in trigeminal and facial nerve ganglia. In four cases the same virus was present both in trigeminal and facial nerve ganglia.

**Figure 1:**
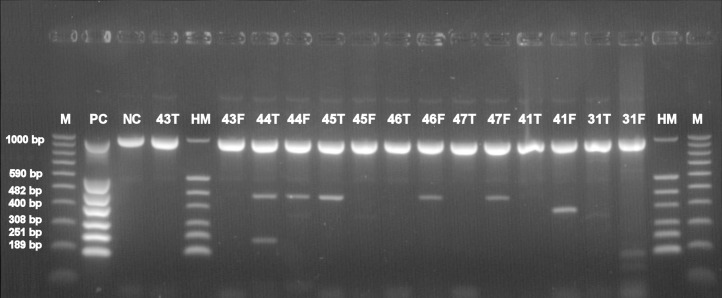
Electrophoretical detection of amplification of herpesviral PCR products from combined right and left facial and trigeminal nerve ganglia on agarose gel. M100-1,000 bp, molecular weight marker; HM, herpes molecular weight marker: 1,000 bp (IC-control standard), 590 bp (CMV), 482 bp (HHV-6), 400 bp (EBV), 308 bp (HSV-2), 251 bp (VZV), 189 bp (HSV-1); PC, positive control; NC, negative control; F, combined samples from right and left facial nerve ganglia; T, combined samples from right and left trigeminal nerve ganglia; Positive sample lines (corresponding to cadaver number): 44T (VZV, HHV-6), 44F(HHV-6), 45T(HHV-6), 46F (HHV-6), 47F (HHV-6), 41F (EBV), 31F(HSV-1).

**Figure 2:**
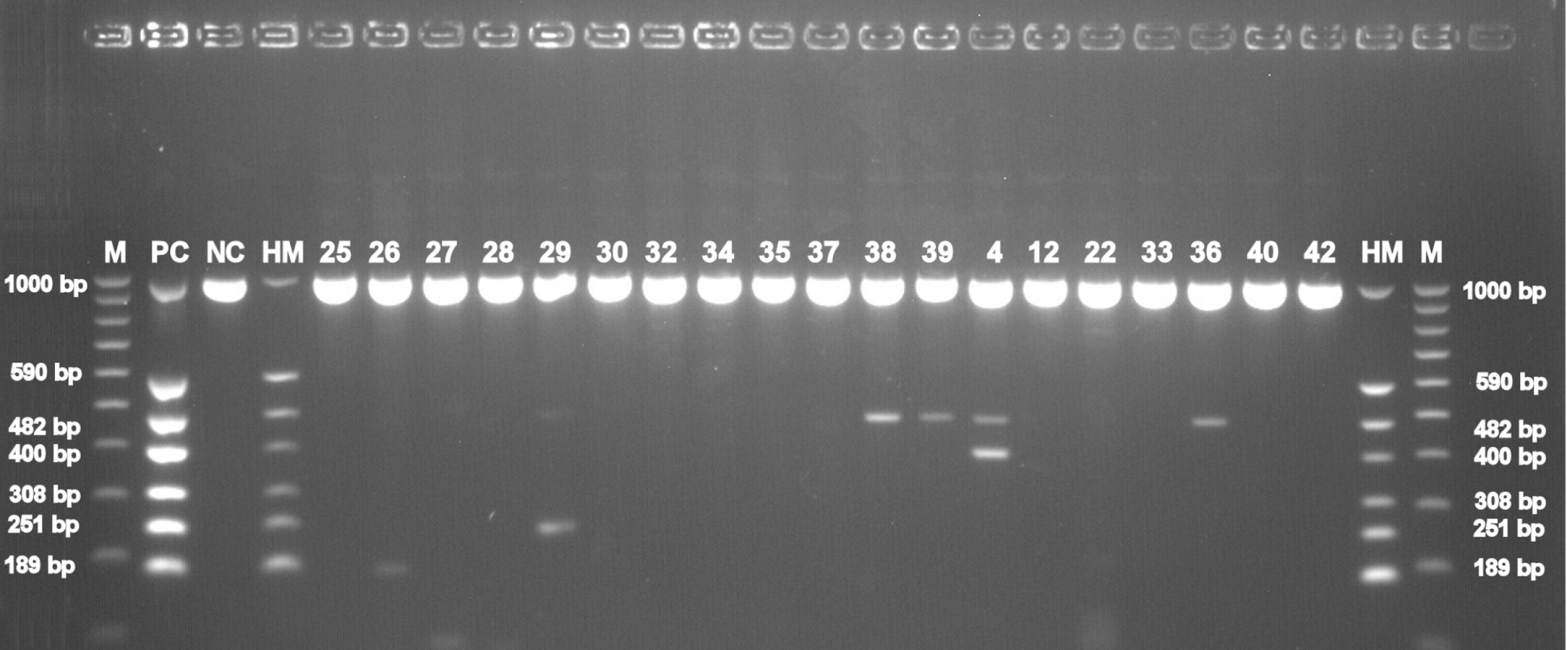
Electrophoretical detection of amplification of herpesviral PCR products from combined right and left trigeminal nerve ganglia on agarose gel. M100-1,000 bp, molecular weight marker; HM, herpes molecular weight marker: 1,000 bp (IC-control standard), 590 bp (CMV), 482 bp (HHV-6), 400 bp (EBV), 308 bp (HSV-2), 251 bp (VZV), 189 bp (HSV-1); PC, positive control; NC, negative control; Positive sample lines (corresponding to cadaver number): 26 (HSV-1), 29 (VZV, HHV-6), 38 (HHV-6), 39 (HHV-6), 4 (EBV, HHV6), 36 (HHV-6).

**Figure 3:**
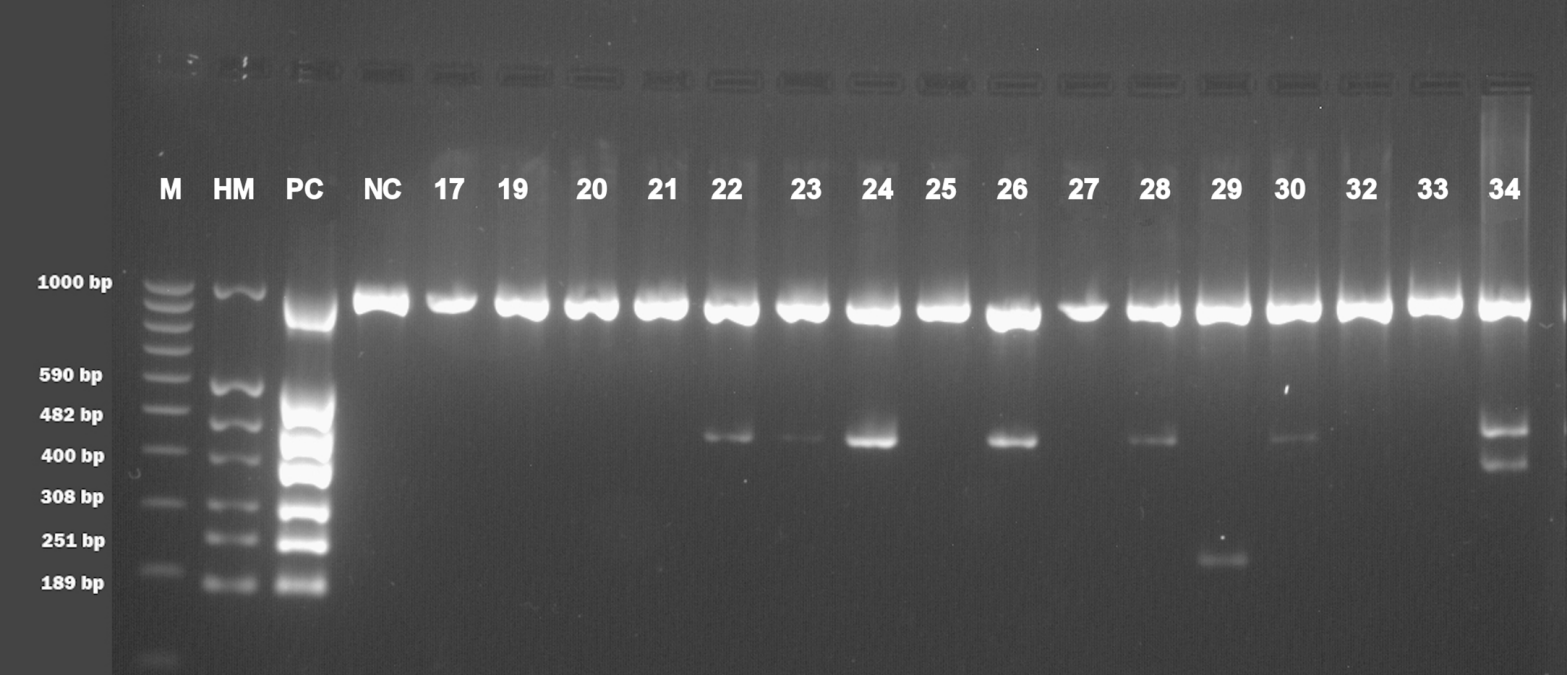
Electrophoretical detection of amplification of herpesviral PCR products from combined right and left facial nerve ganglia on agarose gel. M100-1,000 bp, molecular weight marker; HM, herpes molecular weight marker: 1,000 bp (IC-control standard), 590 bp (CMV), 482 bp (HHV-6), 400 bp (EBV), 308 bp (HSV-2), 251 bp (VZV), 189 bp (HSV-1); PC, positive control; NC, negative control; Positive sample lines (corresponding to cadaver number): 22 (HHV-6), 23 (HHV-6), 24 (HHV-6), 26 (HHV-6), 28 (HHV-6), 29 (VZV), 30 (HHV-6), 34 (EBV, HHV-6).

**Figure 4:**
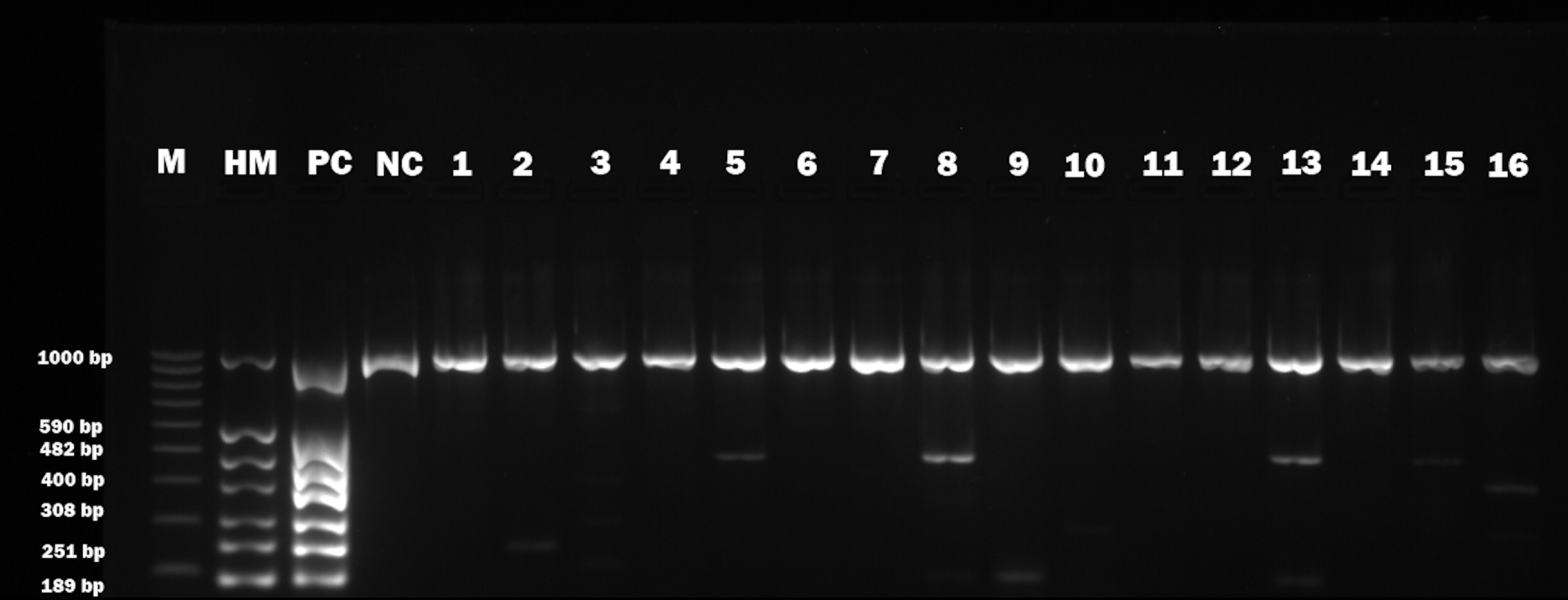
Electrophoretical detection of amplification of herpesviral PCR products from combined right and left facial nerve ganglia on agarose gel. M100-1,000 bp, molecular weight marker; HM, herpes molecular weight marker: 1,000 bp (IC-control standard), 590 bp (CMV), 482 bp (HHV-6), 400 bp (EBV), 308 bp (HSV-2), 251 bp (VZV), 189 bp (HSV-1); PC, positive control; NC, negative control; Positive sample lines (corresponding to cadaver number): 2 (VZV), 5 (HHV-6), 8 (HSV-1, HHV-6), 9 (HSV-1), 10 (HSV-2), 13 (HSV-1, HHV-6), 15 (HHV-6), 16 (HSV-2, EBV).

**Figure 5:**
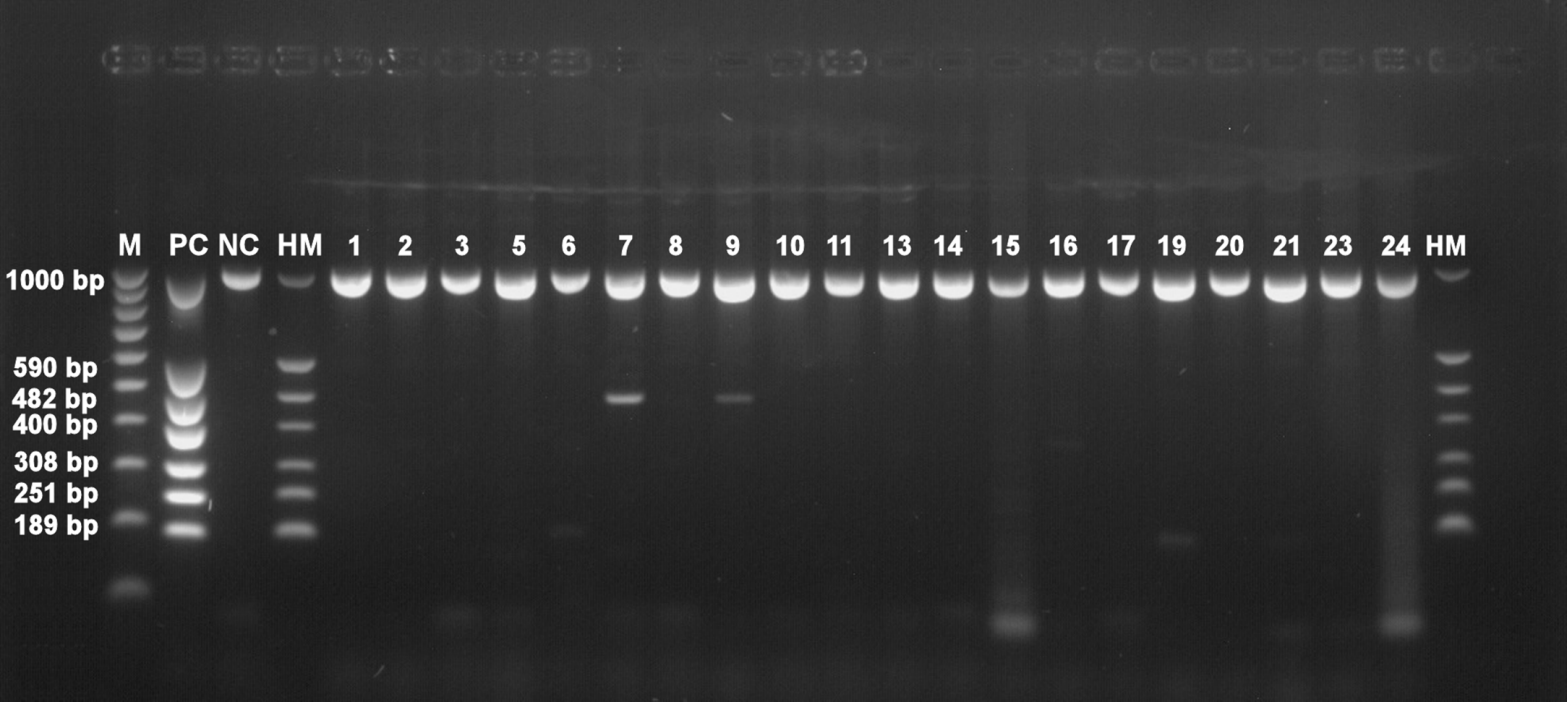
Electrophoretical detection of amplification of herpesviral PCR products from combined right and left trigeminal nerve ganglia on agarose gel. M100-1,000 bp, molecular weight marker; HM, herpes molecular weight marker: 1,000 bp (IC-control standard), 590 bp (CMV), 482 bp (HHV-6), 400 bp (EBV), 308 bp (HSV-2), 251 bp (VZV), 189 bp (HSV-1); PC, positive control; NC, negative control; Positive sample lines (corresponding to cadaver number): 6 (HSV-1), 7 (HHV-6), 9 (HHV-6), 16 (EBV), 19 (HSV-1).

**Figure 6:**
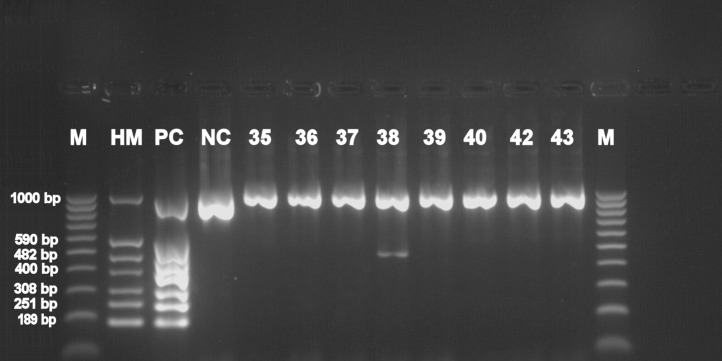
Electrophoretical detection of amplification of herpesviral PCR products from combined right and left facial nerve ganglia on agarose gel. M100-1,000 bp, molecular weight marker; HM, herpes molecular weight marker: 1,000 bp (IC-control standard), 590 bp (CMV), 482 bp (HHV-6), 400 bp (EBV), 308 bp (HSV-2), 251 bp (VZV), 189 bp (HSV-1); PC, positive control; NC, negative control; Positive sample lines (corresponding to cadaver number): 38 (HHV-6).

**Figure 7:**
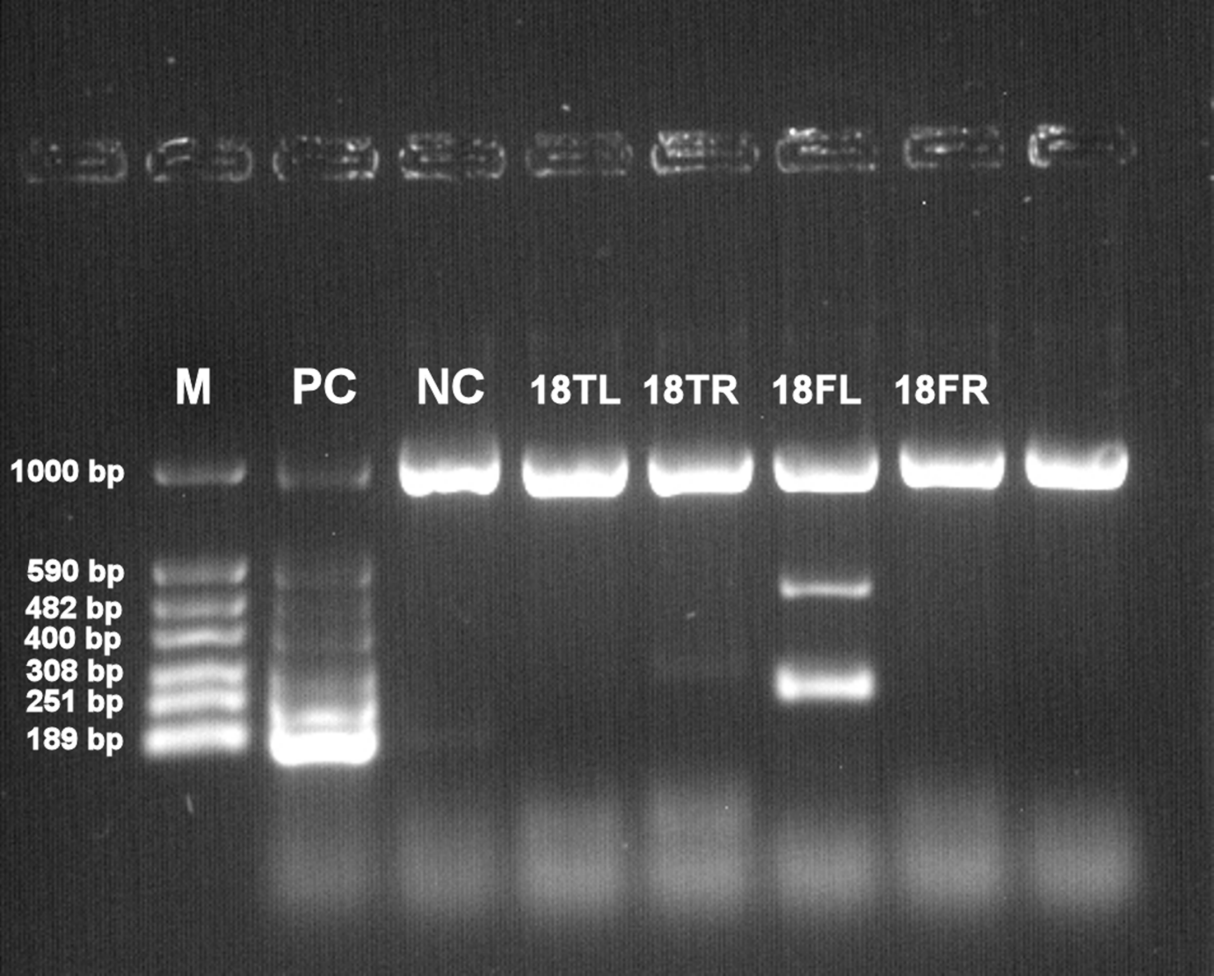
Electrophoretical detection of amplification of herpesviral PCR products from separate right and left trigeminal and facial nerve ganglia on agarose gel. M100-1,000 bp, molecular weight marker; HM, herpes molecular weight marker: 1,000 bp (IC-control standard), 590 bp (CMV), 482 bp (HHV-6), 400 bp (EBV), 308 bp (HSV-2), 251 bp (VZV), 189 bp (HSV-1); PC, positive control; NC, negative control; T, trigeminal nerve ganglia; F, facial nerve ganglia; R, right; L, left; Positive sample lines (corresponding to cadaver number): 18FL (VZV, CMV).

Basic information about cadavers and presence of herpesviruses in trigeminal and facial ganglia of each individual are provided in Table 1. Prevalence of each herpesvirus in trigeminal and facial ganglia is shown in Table 2. The results of electrophoretic detection of herpesviruses PCR products amplification are shown in Figs. 1–7.

**Table 1:** Cadaver information and presence of herpesviruses in trigeminal and facial nerve ganglia.

Identification number	Age (years)	Sex	Cause of death	Trigeminal nerve ganglia	Facial nerve ganglia
2	50	M	Drowning		VZV
4	36	M	Falling from height	EBV, HHV-6	
5	43	M	Sudden death		HHV-6
6	60	M	Sudden death	HSV-1	
7	68	M	Ethanol poisoning	HHV-6	
8	58	M	Ethanol poisoning		HSV-1, HHV-6
9	62	M	Myocardial infarction	HHV-6	HSV-1
10	59	M	Hanging		HSV-2
13	64	M	Cardiac arrhythmia		HSV-1, HHV-6
15	53	M	Hanging		HHV-6
16	41	M	Myocardial infarction	EBV	HSV-2, EBV
18	54	M	Drowning		VZV, CMV
19	31	M	Traffic accident	HSV-1	
22	40	M	Methanol poisoning		HHV-6
23	52	M	Ethanol poisoning		HHV-6
24	46	M	Sudden death		HHV-6
26	35	M	Acetone poisoning	HSV-1	HHV-6
28	38	M	Chest stab wound		HHV-6
29	70	M	Myocardial infarction	VZV, HHV-6	VZV
30	65	M	Ethanol poisoning		HHV-6
31	63	M	Hanging		HSV-1
34	56	M	Hanging		EBV, HHV-6
36	37	M	Traffic accident	HHV-6	
38	28	M	Drowning	HHV-6	HHV-6
39	28	M	Traffic accident	HHV-6	
41	65	M	Hanging		EBV
44	84	F	CO poisoning	VZV, HHV-6	HHV-6
45	54	F	Hanging	HHV-6	
46	26	F	Hanging		HHV-6
47	43	F	Traffic accident		HHV-6

**Table 2:** Prevalence of herpesviruses in trigeminal and facial nerve ganglia of cadavers.

Herpesvirus	Prevalence in cadavers: 30/47 (63.8%)		Prevalence in trigeminal ganglia: 13/47 (27.7%)			Prevalence in facial ganglia: 23/47 (48.9%)		
HSV-1	7/47	14.9%	HSV-1	3/47	6.4%	HSV-1	4/47	8.5%
HSV-2	2/47	4.3%	HSV-2	0/47	0	HSV-2	2/47	4.3%
VZV	4/47	8.5%	VZV	2/47	4.3%	VZV	3/47	6.4%
EBV	4/47	8.5%	EBV	2/47	4.3%	EBV	3/47	6.4%
CMV	1/47	2.1%	CMV	0/47	0	CMV	1/47	2.1%
HHV-6	22/47	46.8%	HHV-6	9/47	19.2%	HHV-6	15/47	31.9%

## Discussion

*Herpesviridae* is a large group of enveloped, double-strained DNA viruses. The characteristic feature of all herpesviruses is their ability to stay dormant within a cell generating a lifelong latent infection in their hosts (Bloom, Giordani & Kwiatkowski, 2010). Herpesviruses are widespread among the human population. Wide variation in seroprevalence of herpesviruses has been observed in different European countries which can be the result of varying population densities, social mixing and climate differences (Galdiero et al., 2018). Some studies suggest that seroprevalence of certain herpesviruses may be higher in urban populations than in the rural populations, while others do not support this finding (Lolekha et al., 2001; Cunningham et al., 2006; Siennicka et al., 2009). In a study carried out by Smith et al. (2006), the overall seropositivity rate for HSV-1 and HSV-2 in four geographic regions in Poland was 90.4% and 9.3%, respectively. Siennicka et al. (2009) reported that seropositivity rate for VZV was approximately 98% in a group of 19-year-olds from Poland. The same author conducted a study among women of childbearing age, where the overall anti-CMV IgG seropositivity was 81.9% (Siennicka et al., 2016). Data regarding seroprevalence of other herpesviruses in general population in Poland is limited.

HSV-1 is widespread throughout the world and establishes latency in sensory ganglia. In the study conducted by Saitoh et al. (2013), the trigeminal ganglia obtained from 12 human cadavers submitted for forensic autopsy within 1–5 days of death were cultured over a layer of Vero cells in order to evaluate whether the virus in the ganglia of the cadavers could be reactivated. It was demonstrated that viable HSV-1 persists in human trigeminal ganglia for some time after death. The presence of HSV-1 detected by PCR in trigeminal human ganglia was found by Motani et al. (2006) in almost 65% of 207 human cadavers and in 60% of these cadavers, the presence was bilateral. In a study conducted by Richter et al. (2009), the distribution of HSV-1 in head and neck ganglia was evaluated and presence of the virus was detected in trigeminal, facial, and many other, both sensory and autonomic, ganglia. Overall, 36% of the analyzed samples were positive for HSV-1 DNA in this study. The results suggest that HSV-1 latency extends beyond the trigeminal nerve ganglia and the virus may reactivate from non-trigeminal site causing atypical presentation. In research carried out by Hill et al. (2008), HSV-1 was found in 155/174 (90.1%) trigeminal ganglia of investigated subjects. Vrabec & Payne (2001) studied trigeminal, geniculate, vestibular, spiral, and vagal ganglia of 18 cadavers and detected HSV DNA in 42% of surveyed ganglia. The aforementioned studies, similarly to our study, were performed on random individuals with no active HSV infection at the time of death. In our study, HSV-1 was found both in the facial nerve ganglia and trigeminal ganglia. HSV-1 DNA was present in 7/47 (14.9%) of cadavers. Furthermore, simultaneous presence of HSV-1 and HHV-6 in the same or different ganglia was found in three cadavers in our study.

HSV-2 reactivates predominantly from lumbar-sacral ganglia giving rise to recurrent genital disease (Margolis et al., 2007; Lafferty et al., 1987). However, HSV-2 has been also implicated in oral-labial infections and may also cause meningitis or encephalitis (Lafferty et al., 1987; Moon et al., 2014). In a study carried out by Richter et al. (2009), superior cervical ganglion, nodose ganglion, pterygopalatine ganglion, ciliary ganglion, otic ganglion, submandibular ganglion, and geniculate ganglion did not manifest any trace of HSV-2. In the present study, HSV-2 DNA was found in two samples of facial nerve ganglia but was not detected in trigeminal ganglia. In one cadaver, HSV-2 DNA coexisted with EBV DNA detected in trigeminal and facial nerve ganglia.

Varicella zoster virus establishes latency in the sensory ganglia after primary infection and the viral DNA is found mainly in trigeminal, geniculate, and thoracic ganglia. Human trigeminal ganglia are commonly dually infected with HSV-1 and VZV (Theil et al., 2002). Numerous studies were undertaken to assess VZV prevalence in the head and neck ganglia. In a study carried out by Inoue et al. (2010), VZV was detected in 94.4% (in 97.1% cadavers) of trigeminal ganglia and HSV-1 was found in 60.6% of trigeminal ganglia (in 64.7% cadavers). In an examination of human trigeminal ganglia performed by Cohrs, Barbour & Gilden (1996), VZV was detected in all samples and HSV-1 was found in 72.7% samples from 25 cadavers. In a study conducted by Richter et al. (2009) 22/36 (61%) of cadavers had VZV DNA in at least one ganglion and overall, there were more HSV-1-positive samples than VZV-positive samples (36% vs. 18%). In our study, HSV-1 DNA was found in more samples than VZV, both in trigeminal and facial nerve ganglia. In a study carried out by Vrabec & Payne (2001), VZV was detected in 64/144 (44%) of studied cranial nerve ganglia. Furuta et al. (1992) detected VZV DNA in 9/13 (69.2%) of examined geniculate ganglia and 11/14 (78.6%) of trigeminal ganglia of adults with no history of recent infection (chickenpox or shingles). In the subsequent study by the same author, VZV DNA was detected in 2/10 (20%) of vestibular ganglia and in 2/10 (20%) of spiral ganglia from five adults (Furuta et al., 1997). In our study VZV was detected in 4 (8.5%) of cadavers.

After primary infection, EBV establishes latency within B-cell pool. Beyond mononucleosis, the best known clinical manifestation of EBV, other conditions have been associated with primary infection, such as hematologic abnormalities or neurologic syndromes (Tselis et al., 1997; Patil et al., 2012; Celik et al., 2015). Although EBV has been linked mostly with lymphoproliferative disease in immunosuppression (such as Burkitt’s lymphoma, Hodgkin’s disease, T-cell lymphomas, nasopharyngeal carcinoma), the virus can affect virtually any organ and cause a wide range of disease manifestations (e.g., pneumonia, myocarditis, pancreatitis, mesenteric adenitis, myositis, and glomerulonephritis) (Young & Murray, 2003). Cases of encephalitis, encephalomyelitis, chronic meningitis, and central nervous system vasculopathy due to EBV infection have been reported (Tselis et al., 1997; Patil et al., 2012; Celik et al., 2015). Bell’s palsy is most commonly associated with HSV and VZV, but other *Herpesviriade*, such as EBV and CMV may also cause acute peripheral facial palsy (McFarlin & Peckler, 2008). Moreover, EBV has been linked to other neurological pathologies, including multiple sclerosis, primary central nervous system lymphoma, Alzheimer’s disease, and cerebellar ataxia. The association of these diseases with EBV was largely based on the presence of high EBV titers in cerebrospinal fluid samples from the patients. However, there is evidence that EBV can directly infect neurons and therefore may be a potential factor in the pathogenesis of these conditions. In in vitro studies, EBV undergoes a predominantly lytic replication in neuronal cells (Jha et al., 2015), but perhaps it may also establish latency in neurons. We have not found any studies describing presence of EBV in trigeminal or facial ganglia. In our study, EBV was present in 4/47 (8.5%) cadavers in trigeminal and/or facial ganglia.

Cytomegalovirus is a common virus with seroprevalence rates varying from 45% to 100% of the adult population (Cannon, Schmid & Hyde, 2010). CMV infection is also the most frequent congenital viral infection which has been associated with permanent neurological impairment, including cerebral palsies, sensorineural hearing loss and neurodevelopmental abnormalities. Primary CMV infection is followed by viral latency and possibility of reactivation (Chavanas, 2016). CMV is known to cause encephalitis and multiple cranial nerve palsies in immunocompromised (Karna et al., 2001). Studies investigating permissiveness of neurons to CMV infection in vitro have lead to conflicting results. Although primary mature human neurons showed no antigen expression or cytopathic effect after in vitro infection, neurons derived from human neural progenitor cells prepared from fetus have been reported to be permissive to CMV infection in some studies. Furthermore, it has been demonstrated that murine CMV is able to establish latency in neural stem progenitor cells and reactivate in mouse brain slice culture (Chavanas, 2016). In our study, CMV was present in the facial nerve ganglia in one cadaver but was absent in trigeminal ganglia.

The primary HHV-6 infection of adults is relatively rare. The virus infects mostly children in the first two years of life, presenting as roseola (Zerr et al., 2005). Seroprevalence rate of HHV-6 ranges from 52% to over 90% in some studies (Cone et al., 1993). HHV-6 has been demonstrated to be neurotropic and is able to persist in a latent form in the nervous system, where it can reactivate, causing wide spectrum of neurological manifestations. Since its discovery, HHV-6 has been implicated in the development of a wide array of neurological conditions, such as seizures, encephalitis, mesial temporal lobe epilepsy, and multiple sclerosis. In recent years HHV-6 has become an increasingly recognized cause of post-transplant limbic encephalitis. It has also been associated with encephalitis in adult immunocompetent patients and is likely to be underdiagnosed as a cause of encephalitis (Ogata, Fukuda & Teshima, 2015; Yilmaz et al., 2018).

In a study conducted by Harberts et al. (2011), distribution of HHV-6 in different anatomical locations of central nervous system was evaluated. The authors have demonstrated that frequency of HHV-6 in the olfactory bulb/tract (50%) was among the highest in the brain regions examined. Furthermore, they confirmed that nasal cavity is a reservoir of HHV-6 and that specialized olfactory-ensheathing glial cells located within the nasal cavity support HHV-6 replication in vitro, which provides evidence for the hypothesis that HHV-6 may enter the central nervous system via olfactory pathway. Hüfner et al. (2007) detected HHV-6 in 30% of trigeminal, 40% of geniculate, 25% of vestibular, and 55% of dorsal root ganglia. Furthermore, in their research, HHV-6 was found to co-occur with HSV-1 or VZV in 91% of ganglia. In a study conducted by Chapenko et al. (2016), the authors evaluated frequency of HHV-6 and HHV-7 genomic sequences in central nervous system DNA samples from post-mortem individuals with unspecified encephalopathy. A significantly higher frequency of single HHV-6 and HHV-6 with HHV-7 was found in pia mater meninges, frontal lobe, temporal lobe, and olfactory tract DNA in individuals with encephalopathy than in a control group. Among all of the viruses included in our study, HHV-6 was the most commonly detected virus and was found in almost half of cadavers (46.8%) with a predominance in the facial nerve ganglia. Other herpesviruses (VZV, EBV, HSV-1) have been found to co-occur with HHV-6 in 6/23 (26.1%) samples of the HHV-6 positive ganglia. HHV-6 is possibly one of the first herpesviruses to infect the ganglia and it has been speculated whether latent HHV-6 infection of the ganglia could create an environment which facilitates the invasion of the other herpesviruses (Hüfner et al., 2007).

The limitation of this study was a relatively small sample size, which could be enlarged in further studies for better assessment of herpesviruses’ distribution in nerve ganglia.

## Conclusions

The results of this study have revealed a common presence of latent form of six herpesviruses among a random group of Polish population. All of the herpesviruses described in this article have neurotropic potential. Surprisingly, the presence of the viral DNA was observed in higher frequency in facial nerve ganglia than in trigeminal ganglia (48.9% vs. 27.7%). HHV-6 was the most prevalent of all herpesviruses in our study. The data also demonstrated simultaneous infection of the ganglia with different herpesviruses. Although presence of HSV-1, HSV-2, and VZV in various nerve ganglia has been well described, a limited number of studies have been undertaken to assess the presence of EBV, CMV, and HHV-6 in cranial nerve ganglia. This study has contributed to the scope of observations made in relation to the frequency and localization of herpesviruses in different structures of the nervous system.

## Supplemental Information

10.7717/peerj.6095/supp-1 Supplemental Information 1 Table representing basic information about cadavers and distribution of herpesviruses in trigeminal and facial nerve ganglia. TG stands for trigeminal nerve ganglia and FG stands for facial nerve ganglia.

## Additional Information and Declarations

### Data Availability

The following information was supplied regarding data availability: The raw data is provided in Supplementary File.

## References

[ref-1] Bloom DC, Giordani NV, Kwiatkowski DL (2010). Epigenetic regulation of latent HSV-1 gene expression. Biochimica et Biophysica Acta (BBA)—Gene Regulatory Mechanisms.

[ref-2] Bradshaw MJ, Venkatesan A (2016). Herpes simplex virus-1 encephalitis in adults: pathophysiology, diagnosis, and management. Neurotherapeutics.

[ref-3] Cannon MJ, Schmid DS, Hyde TB (2010). Review of cytomegalovirus seroprevalence and demographic characteristics associated with infection. Reviews in Medical Virology.

[ref-4] Celik T, Celik U, Tolunay O, Komur M, Baspinar H, Yilmaz C, Mert G, Yildizdas D (2015). Epstein-Barr virus encephalitis with substantia nigra involvement. Journal of Pediatric Neurosciences.

[ref-5] Chapenko S, Roga S, Skuja S, Rasa S, Cistjakovs M, Svirskis S, Zaserska Z, Groma V, Murovska M (2016). Detection frequency of human herpesviruses-6A, -6B, and -7 genomic sequences in central nervous system DNA samples from post-mortem individuals with unspecified encephalopathy. Journal of NeuroVirology.

[ref-6] Chavanas S (2016). New insights about congenital infection by human cytomegalovirus: unveiling the role of PPARγ. Journal of Infectious Diseases & Therapy.

[ref-7] Cohrs RJ, Barbour M, Gilden DH (1996). Varicella-zoster virus (VZV) transcription during latency in human ganglia: detection of transcripts mapping to genes 21, 29, 62, and 63 in a cDNA library enriched for VZV RNA. Journal of Virology.

[ref-8] Cone RW, Huang ML, Ashley R, Corey L (1993). Human herpesvirus 6 DNA in peripheral blood cells and saliva from immunocompetent individuals. Journal of Clinical Microbiology.

[ref-9] Cunningham AL, Taylor R, Taylor J, Marks C, Shaw J, Mindel A (2006). Prevalence of infection with herpes simplex virus types 1 and 2 in Australia: a nationwide population based survey. Sexually Transmitted Infections.

[ref-10] Furuta Y, Takasu T, Fukuda S, Sato-Matsumura KC, Inuyama Y, Hondo R, Nagashima K (1992). Detection of varicella-zoster virus DNA in human geniculate ganglia by polymerase chain reaction. Journal of Infectious Diseases.

[ref-11] Furuta Y, Takasu T, Suzuki S, Fukuda S, Inuyama Y, Nagashima K (1997). Detection of latent varicella-zoster virus infection in human vestibular and spiral ganglia. Journal of Medical Virology.

[ref-12] Galdiero E, Crudele V, Della Rocca MT, Melardo C, Di Lella FM, Galdiero M, Franci G (2018). Seroprevalence of Herpes viruses in a retrospective study in Southern Italy. Archives of Clinical Microbiology.

[ref-13] Gilden D, Cohrs RJ, Mahalingam R, Nagel MA (2010). Neurological disease produced by varicella zoster virus reactivation without rash. Current Topics in Microbiology and Immunology.

[ref-14] Harberts E, Yao K, Wohler JE, Maric D, Ohayon J, Henkin R, Jacobson S (2011). Human herpesvirus-6 entry into the central nervous system through the olfactory pathway. Proceedings of the National Academy of Sciences of the United States of America.

[ref-15] Hill JM, Ball MJ, Neumann DM, Azcuy AM, Bhattacharjee PS, Bouhanik S, Clement C, Lukiw WJ, Foster TP, Kumar M, Kaufman HE, Thompson HW (2008). The high prevalence of herpes simplex virus type 1 DNA in human trigeminal ganglia is not a function of age or gender. Journal of Virology.

[ref-16] Hüfner K, Arbusow V, Himmelein S, Derfuss T, Sinicina I, Strupp M, Brandt T, Theil D (2007). The prevalence of human herpesvirus 6 in human sensory ganglia and its co-occurrence with alpha-herpesviruses. Journal of Neurovirology.

[ref-17] Inoue H, Motani-Saitoh H, Sakurada K, Ikegaya H, Yajima D, Hayakawa M, Sato Y, Otsuka K, Kobayashi K, Nagasawa S, Iwase H (2010). Detection of varicella-zoster virus DNA in 414 human trigeminal ganglia from cadavers by the polymerase chain reaction: A comparison of the detection rate of varicella-zoster virus and herpes simplex virus type 1. Journal of Medical Virology.

[ref-18] Jha HC, Mehta D, Lu J, El-Naccache D, Shukla SK, Kovacsics C, Kolson D, Robertson ES (2015). Gammaherpesvirus infection of human neuronal cells. mBio.

[ref-19] Karna S, Biswas J, Kumarasamy N, Sharma P, Solomon S (2001). Multiple cranial nerve palsy in an HIV-positive patient. Indian Journal of Ophthalmology.

[ref-20] Kinchington PR, Leger AJS, Guedon J-MG, Hendricks RL (2012). Herpes simplex virus and varicella zoster virus, the house guests who never leave. Herpesviridae.

[ref-21] Lafferty WE, Coombs RW, Benedetti J, Critchlow C, Corey L (1987). Recurrences after oral and genital herpes simplex virus infection. Influence of site of infection and viral type. New England Journal of Medicine.

[ref-22] Lolekha S, Tanthiphabha W, Sornchai P, Kosuwan P, Sutra S, Warachit B, Chup-Upprakarn S, Hutagalung Y, Weil J, Bock HL (2001). Effect of climatic factors and population density on varicella zoster virus epidemiology within a tropical country. American Journal of Tropical Medicine and Hygiene.

[ref-23] Malaiya R, Patel S, Snowden N, Leventis P (2015). Varicella vaccination in the immunocompromised. Rheumatology.

[ref-24] Margolis TP, Imai Y, Yang L, Vallas V, Krause PR (2007). Herpes simplex virus type 2 (HSV-2) establishes latent infection in a different population of ganglionic neurons than HSV-1: role of latency-associated transcripts. Journal of Virology.

[ref-25] McFarlin A, Peckler B (2008). An unusual presentation of *Bell’s palsy*: a case report and review of literature. Journal of Emergencies, Trauma and Shock.

[ref-26] Meyding-Lamadé U, Strank C (2012). Herpesvirus infections of the central nervous system in immunocompromised patients. Therapeutic Advances in Neurological Disorders.

[ref-27] Moon SM, Kim T, Lee EM, Kang JK, Lee S-A, Choi S-H (2014). Comparison of clinical manifestations, outcomes and cerebrospinal fluid findings between herpes simplex type 1 and type 2 central nervous system infections in adults. Journal of Medical Virology.

[ref-28] Motani H, Sakurada K, Ikegaya H, Akutsu T, Hayakawa M, Sato Y, Yajima D, Sato K, Kobayashi K, Iwase H (2006). Detection of herpes simplex virus type 1 DNA in bilateral human trigeminal ganglia and optic nerves by polymerase chain reaction. Journal of Medical Virology.

[ref-29] Ogata M, Fukuda T, Teshima T (2015). Human herpesvirus-6 encephalitis after allogeneic hematopoietic cell transplantation: what we do and do not know. Bone Marrow Transplantation.

[ref-30] Pantry S, Medveczky P (2017). Latency, integration, and reactivation of human Herpesvirus-6. Viruses.

[ref-31] Patil AKB, Azad ZR, Mathew V, Alexander M (2012). Chronic meningitis and central nervous system vasculopathy related to Epstein Barr virus. Annals of Indian Academy of Neurology.

[ref-32] Richter ER, Dias JK, Gilbert JE, Atherton SS (2009). Distribution of HSV-1 and VZV in ganglia of the human head and neck. Journal of Infectious Diseases.

[ref-33] Saitoh H, Momma Y, Inoue H, Yajima D, Iwase H (2013). Viable herpes simplex virus type 1 and varicella-zoster virus in the trigeminal ganglia of human cadavers. Journal of Medical Virology.

[ref-34] Siennicka J, Dunal-Szczepaniak M, Trzcińska A, Godzik P, Rosińska M (2016). High seroprevalence of CMV among women of childbearing age implicates high burden of congenital cytomegalovirus infection in Poland. Polish Journal of Microbiology.

[ref-35] Siennicka J, Trzcińska A, Rosińska M, Litwińska B (2009). Seroprevalence of varicella-zoster virus in Polish population. Przeglad Epidemiologiczny.

[ref-36] Smith JS, Rosińska M, Trzcińska A, Pimenta JM, Litwińska B, Siennicka J (2006). Type specific seroprevalence of HSV-1 and HSV-2 in four geographical regions of Poland. Sexually Transmitted Infections.

[ref-37] Strojnik T, Duh D, Lah TT (2017). Prevalence of neurotropic viruses in malignant glioma and their onco-modulatory potential. In Vivo.

[ref-38] Theil D, Derfuss T, Strupp M, Gilden DH, Arbusow V, Brandt T (2002). Cranial nerve palsies: herpes simplex virus type 1 and varizella-zoster virus latency. Annals of Neurology.

[ref-39] Tselis A, Duman R, Storch GA, Lisak RP (1997). Epstein-Barr virus encephalomyelitis diagnosed by polymerase chain reaction: detection of the genome in the CSF. Neurology.

[ref-40] Vrabec JT, Payne DA (2001). Prevalence of herpesviruses in cranial nerve ganglia. Acta Oto-Laryngologica.

[ref-41] White DW, Beard RS, Barton ES (2012). Immune modulation during latent herpesvirus infection. Immunological Reviews.

[ref-42] Yilmaz M, Yasar C, Aydin S, Derin O, Polat B, Ertan G, Ceylan B, Mert A (2018). Human Herpesvirus 6 encephalitis in an immunocompetent pregnant patient and review of the literature. Clinical Neurology and Neurosurgery.

[ref-43] Young LS, Murray PG (2003). Epstein-Barr virus and *Oncogene*sis: from latent genes to tumours. Oncogene.

[ref-44] Zerr DM, Meier AS, Selke SS, Frenkel LM, Huang M-L, Wald A, Rhoads MP, Nguy L, Bornemann R, Morrow RA, Corey L (2005). A population-based study of primary human herpesvirus 6 infection. New England Journal of Medicine.

